# Material Mapping of QCT-Derived Scapular Models: A Comparison with Micro-CT Loaded Specimens Using Digital Volume Correlation

**DOI:** 10.1007/s10439-019-02312-2

**Published:** 2019-07-11

**Authors:** Nikolas K. Knowles, Jonathan Kusins, Mohammadreza Faieghi, Melissa Ryan, Enrico Dall’Ara, Louis M. Ferreira

**Affiliations:** 1 School of Biomedical Engineering, The University of Western Ontario, London, ON Canada; 2 Department of Mechanical and Materials Engineering, The University of Western Ontario, London, ON Canada; 3 Roth|McFarlane Hand and Upper Limb Centre, St. Josephs Health Care, London, ON Canada; 4 Collaborative Training Program in MSK Health Research, and Bone and Joint Institute, The University of Western Ontario, London, ON Canada; 5 Department of Oncology and Metabolism and INSIGNEO Institute for In Silico Medicine, University of Sheffield, Sheffield, UK; 6 Roth|McFarlane Hand and Upper Limb Centre, Surgical Mechatronics Laboratory, St. Josephs Health Care, 268 Grosvenor St., London, ON Canada

**Keywords:** Finite element modeling, Material mapping strategies, Experimental loading, Bone mechanics

## Abstract

Subject- and site-specific modeling techniques greatly improve finite element models (FEMs) derived from clinical-resolution CT data. A variety of density-modulus relationships are used in scapula FEMs, but the sensitivity to selection of relationships has yet to be experimentally evaluated. The objectives of this study were to compare quantitative-CT (QCT) derived FEMs mapped with different density-modulus relationships and material mapping strategies to experimentally loaded cadaveric scapular specimens. Six specimens were loaded within a micro-CT (33.5 *μ*m isotropic voxels) using a custom-hexapod loading device. Digital volume correlation (DVC) was used to estimate full-field displacements by registering images in pre- and post-loaded states. Experimental loads were measured using a 6-DOF load cell. QCT-FEMs replicated the experimental setup using DVC-driven boundary conditions (BCs) and were mapped with one of fifteen density-modulus relationships using elemental or nodal material mapping strategies. Models were compared based on predicted QCT-FEM nodal reaction forces compared to experimental load cell measurements and linear regression of the full-field nodal displacements compared to the DVC full-field displacements. Comparing full-field displacements, linear regression showed slopes ranging from 0.86 to 1.06, *r*-squared values of 0.82–1.00, and max errors of 0.039 mm for all three Cartesian directions. Nearly identical linear regression results occurred for both elemental and nodal material mapping strategies. Comparing QCT-FEM to experimental reaction forces, errors ranged from − 46 to 965% for all specimens, with specimen-specific errors as low as 3%. This study utilized volumetric imaging combined with mechanical loading to derive full-field experimental measurements to evaluate various density-modulus relationships required for QCT-FEMs applied to whole-bone scapular loading. The results suggest that elemental and nodal material mapping strategies are both able to simultaneously replicate experimental full-field displacements and reactions forces dependent on the density-modulus relationship used.

**Electronic supplementary material:**

The online version of this article (doi:10.1007/s10439-019-02312-2) contains supplementary material, which is available to authorized users.

## Introduction

Subject-specific finite element models (FEMs) allow for a variety of biomechanical and clinical conditions to be tested in a highly repeatable manner. The accuracy of these FEMs is improved by accurately mapping density using quantitative computed tomography (QCT) and by choosing a constitutive relationship that relates density to mechanical properties. Although QCT-derived FEMs have become common practice in contemporary computational studies of whole bones, many of the density-modulus relationships used at the whole-bone-level were derived using mechanical loading of small trabecular or cortical bone cores.^12^,^20^ Although it has been shown that these relationships derived for a variety of anatomic locations can replicate the apparent-level properties of glenoid trabecular bone,^18^,^19^ the efficacy in translating these relationships to the whole-bone-level is unknown.

Similarly, trabecular density–modulus relationships are often extrapolated to the entire density range consisting of both trabecular and cortical bone in whole-bone QCT-FEMs. Few studies have assessed the effect of this mapping—or the use of piecewise transitions between trabecular and cortical bone—and none have done so in the shoulder. Beyond the choice of density-modulus relationship, the material mapping strategy also influences model accuracy.^28^ Recent methods have been proposed evaluating elemental and nodal mapping strategies and pre-processing methods to compare the effect of density-modulus relationships and material mapping strategy on the performance of femoral QCT-FEMs.^9^,^11^ Although these validations provide a comprehensive and robust testing methodology, they are limited to comparisons lying on the cortical shell and global stiffness measurements. Additionally, the boundary conditions (BCs) are limited to those measured with load cells, or other external measures. Recent studies on spine segments have found improvements between QCT-FEMs and experimental results when BCs are derived using local displacements measured by DVC.^14^,^15^

Utilizing these recent advancements in the assignment of BCs and robust full-field comparisons provided by DVC, this study compared QCT-FEMs mapped with various density-modulus relationships and material mapping strategies to experimentally loaded scapular models within a micro-CT. Comparisons were performed on the basis of experimental and QCT-FEM reaction forces and full-field displacements to determine the predictive accuracy of the QCT-FEMs and to identify the best modeling approach.

## Materials and Methods

### Specimens and QCT Scanning

Six fresh-frozen cadaveric full arms (3 male; 3 female; mean age: 68 ± 10 years) were scanned with a multi-slice clinical CT-scanner (GE Discovery CT750 HD, Milwaukee, WI, USA) using clinical settings (pixel size: 0.625 mm to 0.668 mm, slice thickness: 0.625 mm, 120 kVp, 200 mA, BONEPLUS). A dipotassium phosphate (K_2_HPO_4_) calibration phantom (QCT Pro, Mindways Software Inc., Austin, TX, USA) was scanned with each specimen to determine specimen-specific QCT-density relationships. The QCT density for each specimen was determined using these relationships applied to the segmented QCT-FEMs prior to material mapping (Mimics v.20.0, Materialise, Leuven, BE) (Table 1 and Supplementary Materials). Following scanning, each scapula was denuded of all soft-tissues and fixed at its medial aspect by potting in polymethylmethacrylate (PMMA). The glenoid surface was then resurfaced to expose the trabecular bone using a hemispherical total shoulder arthroplasty reamer in order to ensure a uniform surface for loading.

**Table 1:** Sex, age and QCT-density of the six specimens tested.

Specimen	Sex	Age (years)	QCT density (g_K2HPO4_/cm^3^)
1	M	80	0.333 ± 0.256 (0.01–1.312)
2	M	73	0.245 ± 0.198 (0.01–1.194)
3	F	62	0.376 ± 0.240 (0.01–1.220)
4	F	52	0.377 ± 0.253 (0.01–1.298)
5	M	74	0.343 ± 0.292 (0.01–1.341)
6	F	64	0.319 ± 0.254 (0.01–1.138)

Values are mean ± SD (range)

### Experimental Loading and MicroCT Scanning

**Figure 1:**
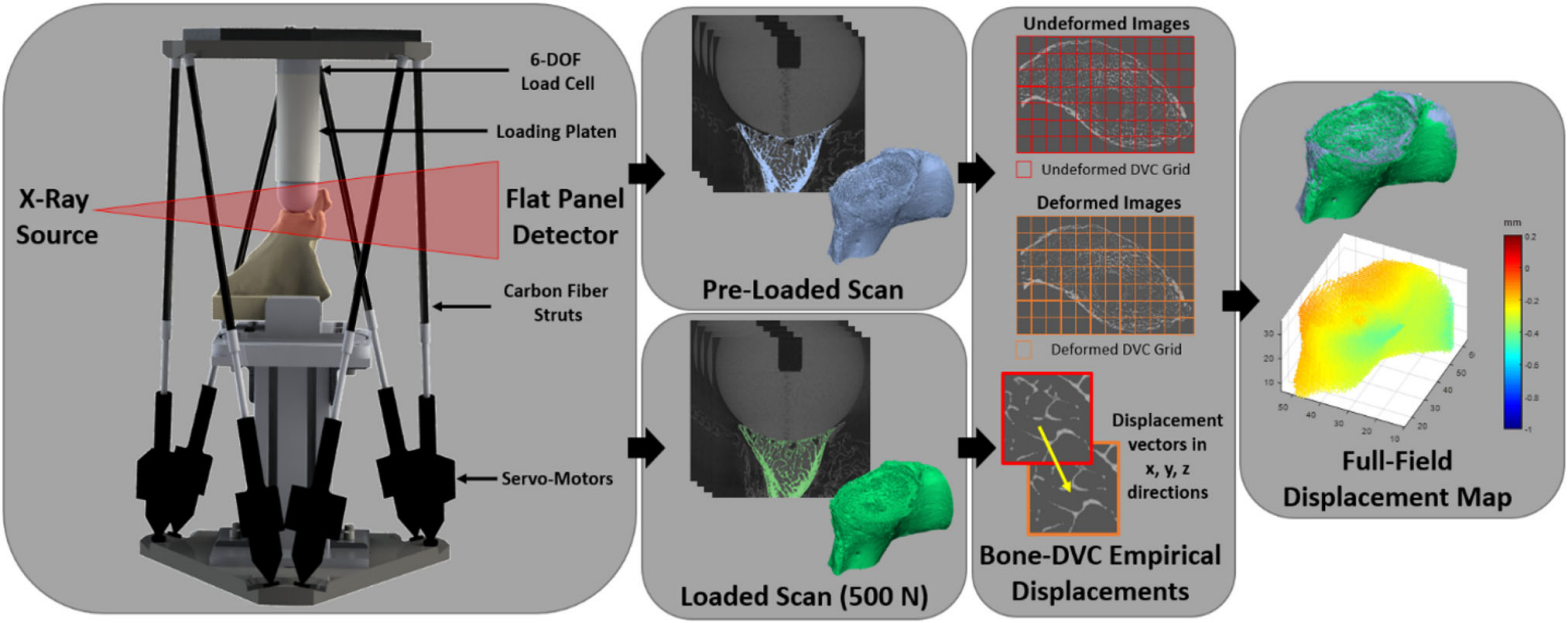
The workflow to determine full-field experimental displacements of cadaveric scapulae. A custom CT-compatible hexapod robot was used to apply compressive loads. Pre- and post-loaded scans were acquired and Bone-DVC 7 was used to compare the two states. An experimental full-field displacement map was used for comparison with the QCT-FEM nodal displacements.

**Figure 2:**
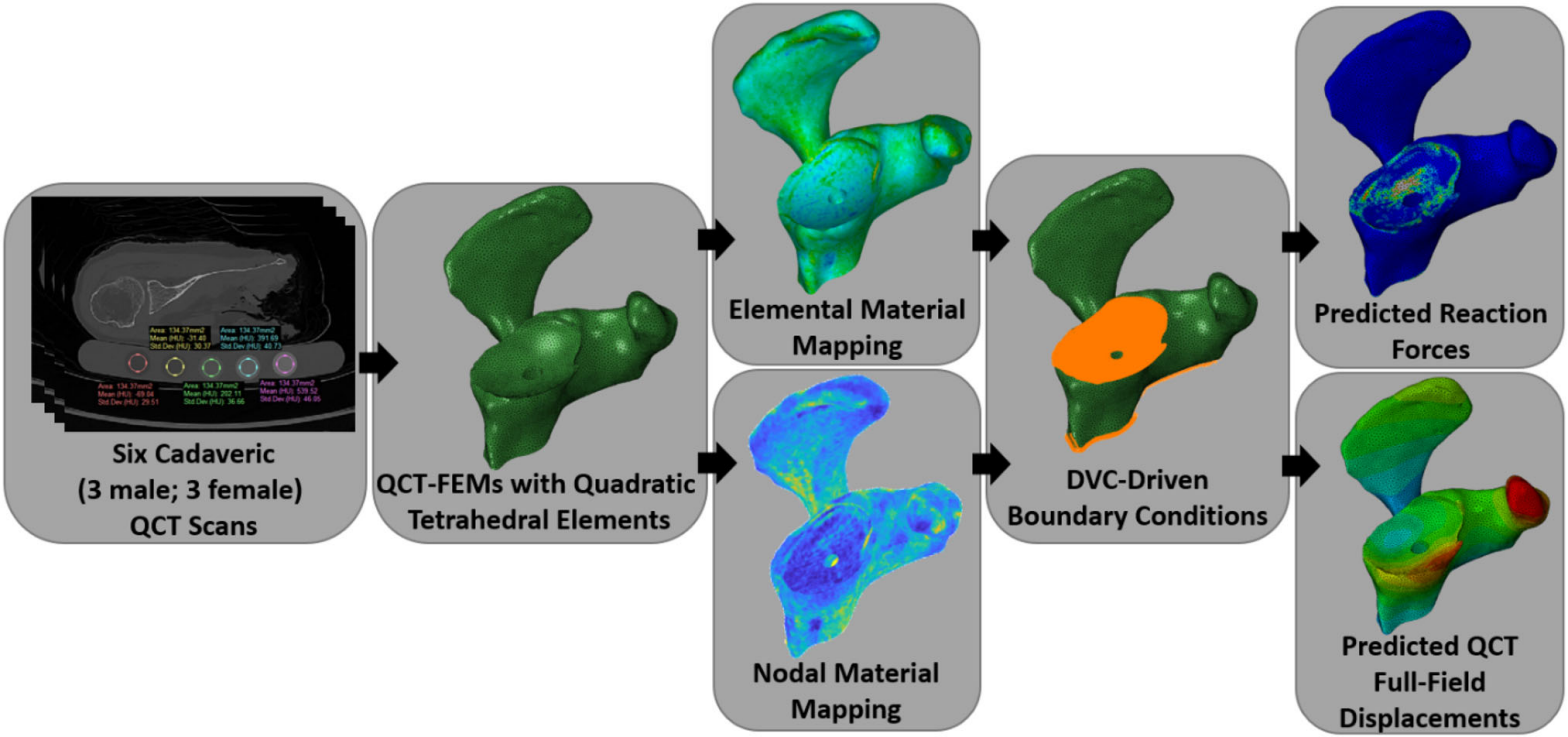
The workflow to determine full-field QCT-FEM displacements and reaction forces of cadaveric scapulae. QCT scans were acquired for six cadaveric specimens using a dipotassium phosphate calibration phantom. These images were used to generate QCT-FEMs with quadratic tetrahedral elements. Each of the fifteen density-modulus relationships (Table 2) were mapped using either elemental or nodal material mapping strategies. DVC-driven boundary conditions were applied to the articular and medial cropped surfaces (orange highlighted nodes). Reaction forces and full-field displacement of QCT-FEMs were compared to experimental DVC.

Each specimen was mounted in a custom hexapod parallel robot designed to apply loads to the glenoid through a 48 mm diameter Delrin® hemisphere (Fig. 1). The hexapod’s six linear servo-motors were augmented with carbon fibre rods to produce a radiolucent section for compatibility with a cone beam scanner and the load applicator was extended with an acrylic cylinder to avoid metal artifact. A 6-degree-of-freedom load cell (Mini 45, ATI Industrial Automation, NC, USA), integrated into the hexapod’s loading platform, was used to target experimental applied loads. The hexapod was placed within a cone-beam microCT scanner (Nikon XT H 225 ST, Nikon Metrology, NV), each specimen was hydrated with phosphate-buffered saline solution, wrapped in saline-soaked tissue and a pre-load of 10 N was applied. Under these conditions, a pre-load scan was acquired (33.5 *μ*m isotropic voxels, 95 kVp, 64 *μ*A, 3141 projections, 1000 ms exposure) after 20 min to allow proper relaxation of the loaded structure. The field of view (FOV) within the microCT varied by specimen, due to size, but included the entire glenoid vault and partial scapular body for all specimens (Fig. 2). Following the pre-loaded scan, a compressive load to a target 500 N was performed. The loaded structure was again allowed to settle for 20 min and a scan with identical settings was performed at this post-loaded state. The load was measured immediately prior to this 52-min scan. Identical loading regimes were performed for all six scapular specimens.

### Image Post-processing and Digital Volume Correlation (DVC)

The pre- and post-loaded scans were post-processed to provide 8-bit images of the bone using a specimen-specific threshold (Mimics v.20.0 & ImageJ).^27^ These images were registered elastically using the Bone-DVC software.^8^ Bone-DVC is a global DVC software that computes a full-field displacement map by superimposing a regular grid with nodal spacing on the undeformed (pre-loaded state) and deformed (post-loaded state) images. The registration equations are solved at the nodes of the grid by assuming linear displacements within each grid cell. An optimised smoothing coefficient is used to regularize the displacement field. This approach was shown to improve the accuracy of bone strain and displacement measurements for different bone structures at different image resolutions.^5^,^7^,^8^ Moreover Bone-DVC has previously been used to validate the outputs of different FEM approaches for trabecular bone,^4^ vertebral bodies,^6^ and mouse tibia.^23^

In the first specimen, two pre-loaded scans were acquired and compared to determine the optimal DVC nodal spacing, with the assumption that no rotations happened between the two repeated scans. Comparison of these scans was performed to quantify accuracy and precision of the displacement measurements as previously described.^7^,^8^ A nodal spacing of 30, equivalent to a sub-volume size of ≈ 1 mm was found to provide the best compromise between the spatial resolution of the displacement measurement and its precision (errors, evaluated as standard deviations of the displacement measurements along each directions for every node, equal to 1.5, 2.0, and 1.1 *µ*m in the *x*, *y*, and *z* direction, respectively). Bone-DVC was used to determine the full-field displacements for all six cadaveric specimens (Fig. 1).

### QCT-FEM Generation and Boundary Conditions

To replicate the DVC-experimental results in subsequent QCT-FEMs, the scapula was cropped to include only the region included in the DVC results. The entire coracoid was included in the QCT-FEMs because our previous studies have shown that removal of this structure greatly influences the loading characteristics of the scapula.^17^ The QCT-FEMs were generated from each corresponding QCT scan that was acquired at clinical resolution. These models were segmented using a global threshold of 225 HU and then filled using embedded semi-automated morphological tools (Mimics v.20.0). The outer bone contours were qualitatively assessed. The model’s glenoid surface was virtually subtracted to match the resurfaced glenoid of each cadaveric specimen. This QCT model was aligned to a 3D model of the experimental scapula using iterative closest points registration (3-matic v.12.0, Materialise, Leuven, BE). This further ensured the geometrical accuracy of the scapular QCT-FEMs. Similar to the co-registration method previously described,^18^ the coordinate transform between the clinical-scans and the micro-CT scans were used to ensure computational forces and displacements matched the experimental setup. A triangular surface mesh of each model was created with a target 1 mm edge length and optimal 60° angles between edges.^2^ Surface meshes were transferred to Abaqus (v.6.14, Simulia, Providence, RI) and meshed with 10-node tetrahedral elements.

To accurately replicate the boundary conditions of each QCT-FEM, DVC-driven BCs were applied on both the articular and the medial cropped surfaces (Fig. 2). Custom Matlab code (v.R2017a, Mathworks, Natick, MA) was used to create these DVC-driven BCs in the Abaqus input file. Tri-linear interpolation of the DVC displacement-field was performed to assign displacement boundary conditions in the *x*, *y*, and *z* directions to the tetrahedral nodes of the medial and glenoid articular surface, using previously described methods.^21^

### Density–Modulus Relationships and Material Mapping Strategies

Fifteen density-modulus relationship combinations were compared with variations in the density ranges of the trabecular and cortical mapping (Table 2). The five primary relationships developed in the literature were derived from trabecular/cortical bone cores (relationships 3, 6, 9, 12, 15). Relationship 15 used a piecewise transition between trabecular and cortical bone of 1.54 g/cm^3^ and was the only one of the primary relationships that had a trabecular/cortical piecewise relationship. This was included as it is a common relationship reported in shoulder FEM studies.^19^ Relationships 1, 4, 7, 10, and 13 used a transition from trabecular to cortical bone at an apparent density of 1.0 g/cm^3^ (QCT equivalent density of 0.453 g_K2HPO4_/cm^3^).^10^ Relationships 2, 5, 8, 11, and 14, assumed a uniform modulus of 20,000 MPa for all bone with an apparent density greater than the mean apparent density of cortical bone (ρ_app_ > 1.8 g/cm^3^; QCT equivalent density of 0.818 g_K2HPO4_/cm^3^).^3^ These fifteen relationships were mapped using either elemental (Mimics v. 20.0) or nodal (Matlab, v.R2017a) material mapping strategies. The former is implemented in commercial software and uses exact volume element averaging of the tetrahedral mesh overlaid on the native CT-scaler field. The latter was implemented in custom code using tri-linear interpolation of the tetrahedral nodal coordinates within the native CT-scaler field. This nodal mapping strategy code also accounted for partial volume effects (PVEs) by assigning surface nodes a modulus equal to the nearest internal nodes, if this node’s modulus was higher than the PVE affected surface node.^13^ In total, there were 90 elemental-mapped QCT-FEMs and 90 nodal-mapped QCT-FEMs for comparison.

**Table 2:** Density–modulus relationships.

	Density range	*ρ*-*E* relationship
1	$\rho_{\text{qct}} < 0.453$ g_K2HPO4_/cm^3^ $\rho_{\text{qct}} \ge 0.453$ g_K2HPO4_/cm^3^	^a^$E_{\text{trab}} = 32,790 \times \rho_{\text{qct}}^{2.307}$ ^b^$E_{\text{cort}} = 10,200 \times \rho_{\text{ash}}^{2.01}$
2	$\rho_{\text{qct}} < 0.818$ g_K2HPO4_/cm^3^ $\rho_{\text{qct}} \ge 0.818$ g_K2HPO4_/cm^3^	^a^$E_{\text{trab}} = 32,790 \times \rho_{\text{qct}}^{2.307}$ $E_{\text{cort}} = 20 ,000 {\text{MPa}}$
3	$\rho_{\text{qct}} < 0.818$ g_K2HPO4_/cm^3^ $\rho_{\text{qct}} \ge 0.818$ g_K2HPO4_/cm^3^	^a^$E_{\text{trab}} = 32,790 \times \rho_{\text{qct}}^{2.307}$ ^a^$E_{\text{cort}} = 32,790 \times \rho_{\text{qct}}^{2.307}$
4	$\rho_{\text{qct}} < 0.453$ g_K2HPO4_/cm^3^ $\rho_{\text{qct}} \ge 0.453$ g_K2HPO4_/cm^3^	^c^$E_{\text{trab}} = 38,780 \times \rho_{\text{qct}}^{1.88}$ ^b^$E_{\text{cort}} = 10,200 \times \rho_{\text{ash}}^{2.01}$
5	$\rho_{\text{qct}} < 0.818$ g_K2HPO4_/cm^3^ $\rho_{\text{qct}} \ge 0.818$ g_K2HPO4_/cm^3^	^c^$E_{\text{trab}} = 38,780 \times \rho_{\text{qct}}^{1.88}$ $E_{\text{cort}} = 20, 000 {\text{MPa}}$
6	$\rho_{\text{qct}} < 0.818$ g_K2HPO4_/cm^3^ $\rho_{\text{qct}} \ge 0.818$ g_K2HPO4_/cm^3^	^c^$E_{\text{trab}} = 38,780 \times \rho_{\text{qct}}^{1.88}$ ^c^$E_{\text{cort}} = 38,780 \times \rho_{\text{qct}}^{1.88}$
7	$\rho_{\text{app}} < 1.0$ g/cm^3^ $\rho_{\text{app}} \ge 1.0$ g/cm^3^	^d^$E_{\text{trab}} = 8920 \times \rho_{\text{app}}^{1.83}$ ^b^$E_{\text{cort}} = 10,200 \times \rho_{\text{ash}}^{2.01}$
8	$\rho_{\text{app}} < 1.8$ g/cm^3^ $\rho_{\text{app}} \ge 1.8$ g/cm^3^	^d^$E_{\text{trab}} = 8920 \times \rho_{\text{app}}^{1.83}$ $E_{\text{cort}} = 20 ,000 {\text{MPa}}$
9	$\rho_{\text{app}} < 1.8$ g/cm^3^ $\rho_{\text{app}} \ge 1.8$ g/cm^3^	^d^$E_{\text{trab}} = 8920 \times \rho_{\text{app}}^{1.83}$ ^d^$E_{\text{cort}} = 8920 \times \rho_{\text{app}}^{1.83}$
10	$\rho_{\text{app}} < 1.0$ g/cm^3^ $\rho_{\text{app}} \ge 1.0$ g/cm^3^	^e^$E_{\text{trab}} = 15,000 \times \left( {\frac{{\rho_{\text{app}} }}{1.8}} \right)^{2}$ ^b^$E_{\text{cort}} = 10,200 \times \rho_{\text{ash}}^{2.01}$
11	$\rho_{\text{app}} < 1.8$ g/cm^3^ $\rho_{\text{app}} \ge 1.8$ g/cm^3^	^e^$E_{\text{trab}} = 15,000 \times \left( {\frac{{\rho_{\text{app}} }}{1.8}} \right)^{2}$ $E_{\text{cort}} = 20 ,000 {\text{MPa}}$
12	$\rho_{\text{app}} < 1.8$ g/cm^3^ $\rho_{\text{app}} \ge 1.8$ g/cm^3^	^e^$E_{\text{trab}} = 15,000 \times \left( {\frac{{\rho_{\text{app}} }}{1.8}} \right)^{2}$ ^e^$E_{\text{cort}} = 15,000 \times \left( {\frac{{\rho_{\text{app}} }}{1.8}} \right)^{2}$
13	$\rho_{\text{app}} < 1.0$ g/cm^3^ $\rho_{\text{app}} \ge 1.0$ g/cm^3^	^f^$E_{\text{trab}} = 60 + 900 \times \rho_{\text{app}}^{2}$ ^b^$E_{\text{cort}} = 10,200 \times \rho_{\text{ash}}^{2.01}$
14	$\rho_{\text{app}} < 1.8$ g/cm^3^ $\rho_{\text{app}} \ge 1.8$ g/cm^3^	^f^$E_{\text{trab}} = 60 + 900 \times \rho_{\text{app}}^{2}$ $E_{\text{cort}} = 20 ,000 {\text{MPa}}$
15	$\rho_{\text{app}} < 1.54$ g/cm^3^ $\rho_{\text{app}} \ge 1.54$ g/cm^3^	^f^$E_{\text{trab}} = 60 + 900 \times \rho_{\text{app}}^{2}$ ^g^$E_{\text{cort}} = 90 \times \rho_{\text{app}}^{7.4}$

Density-modulus relationships are from: ^a,c^Knowles et al.^18^; ^b^Keller et al.^16^;^d^Morgan et al.^22^; ^e^Büchler et al.^1^; ^f^Schaffler and Burr^25^; ^g^Rice et al.^24^ Apparent density $\left( {\rho_{\text{app}} } \right)$ was converted to ash density $\left( {\rho_{\text{ash}} } \right)$ using the relationship $\rho_{\text{ash}} = 0.6\rho_{\text{app}}$^26^

Relationships 1, 4, 7, 10, 13 use a transition between trabecular and cortical material mapping at 0.453 g_K2HPO4_/cm^3^ (1.0 g/cm^3^ apparent density), relationships 2, 5, 8, 11, 14 at 0.818 g_K2HPO4_/cm^3^ (1.8 g/cm^3^ apparent density). Relationship 15 uses a transition at 0.697 g_K2HPO4_/cm^3^ (1.54 g/cm^3^ apparent density), and relationships 3, 6, 9, and 12 use the trabecular density-modulus relationship extrapolated across the entire density range

### QCT and DVC Model Comparisons

The nodal reaction forces were extracted from each QCT-FEM to determine which density-modulus relationship and material mapping strategy most accurately replicated the experimental reaction forces, measured with the load cell. Custom-code (Matlab v. R2017a) summed the reaction forces that occurred at the articular and medial surfaces of the DVC-driven QCT-FEM. The code was used to verify that the QCT-FEM reaction forces were in equilibrium (forces were equal and opposite) and furthermore the sum of predicted forces occurring at the articular surface was compared to the experimental force. The difference between these were plotted as percentage error (Eq. 1) for each of the fifteen density-modulus relationship by specimen. The percentage errors in reaction force were also plotted against mean mapped modulus for the different trabecular and cortical mapping density-modulus relationships.

1 $${\text{\% error}} = \left( {\frac{{\left( {{\text{QCTFEM force}} - {\text{Exp}} . {\text{force}}} \right)}}{{{\text{Exp}} . {\text{force}}}}} \right) \times 100.$$

The QCT-FEM nodal displacements were compared to the full-field experimental DVC displacement results as the gold standard, using linear regression. The QCT-FEM nodes were region averaged within a sub-volume cubic size of 1 mm dependent on the location of the DVC nodal locations before comparing to DVC displacements to account for the increased number of FEM nodes to DVC grid points.^15^ The regions where the displacements were compared were cropped to include only the volume of the scapula included in DVC assessment. The DVC-driven nodes at the BCs were removed from the displacement comparisons, as previously described.^15^ Outliers were removed using the 5 × the Cooks distance method previously described.^6^ Linear regression was used to compare the region averaged QCT-FEM nodal displacement results to the full-field DVC displacement results in the *x* (UX), *y* (UY), and *z* (UZ), directions.

## Results

Nearly identical linear regression results between displacements predicted by QCT-FEMs mapped with elemental or nodal material mapping strategies and experimental DVC measurements (Table 3). The lowest slope was in the y-direction (0.86), which also had the lowest *r*-squared values (0.82). Root mean square error (RMSE) and max error were 0.018 and 0.039 mm for all Cartesian directions, respectively.

**Table 3:** Linear regression results of QCT-FEM and DVC experimental nodal displacement fields.

Displacement direction	Material mapping strategy	Slope	Intercept	*r*^2^	RMSE (mm)	Max error (mm)
UX	Elemental	0.94 to 1.06	− 0.020 to 0.002	0.97 to 1.00	0.003 to 0.013	0.010 to 0.038
	Nodal	0.94 to 1.06	− 0.020 to 0.002	0.97 to 1.00	0.003 to 0.013	0.010 to 0.039
UY	Elemental	0.86 to 1.05	− 0.011 to 0.009	0.82 to 1.00	0.003 to 0.010	0.008 to 0.038
	Nodal	0.86 to 1.04	− 0.012 to 0.010	0.82 to 1.00	0.003 to 0.010	0.007 to 0.036
UZ	Elemental	1.00 to 1.06	− 0.005 to 0.010	0.94 to 1.00	0.003 to 0.018	0.009 to 0.037
	Nodal	1.00 to 1.06	− 0.005 to 0.010	0.94 to 1.00	0.002 to 0.018	0.008 to 0.037

Values are range of six specimens and fifteen density–modulus relationship combinations (*n *= 90 elemental and *n *= 90 nodal QCT-FEMs)

**Figure 3:**
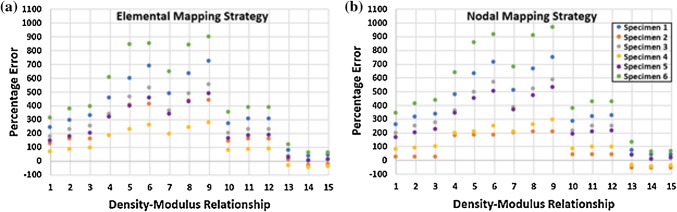
Percentage error plots in reaction force between experimentally loaded scapular specimens and QCT-FEMs generated with 15 different density-modulus relationships (Table 2) and elemental **(a)** or nodal **(b)** material mapping strategies.

The target experimental force magnitude for each specimen was 500 N. The actual measured force magnitudes after relaxation, but prior to scanning for each specimen were 496, 449, 491, 491, 487, and 480 N, for specimens 1 to 6, respectively. The computational reaction forces showed large variation across all specimens and density-modulus relationships when an elemental material mapping strategy was used (Fig. 3a). The percentage error in computational reaction forces ranged from 37 to 719% in specimen 1, − 27 to 439% in specimen 2, 7 to 550% in specimen 3, − 46 to 274% in specimen 4, − 3 to 486% in specimen 5, 57 to 899% in specimen 6. For this material mapping strategy, specimens 1, 3, 5, 6 had the lowest percentage errors, of 37, 7, − 3, and 57% respectively, when relationship 14 was used in the QCT-FEMs. Specimens 2 and 4 had a slightly lower percentage errors of 3 and 38% respectively, when using relationship 13.

Similarly, when using a nodal material mapping strategy (Fig. 3b), there were large variations among specimens when mapped using different material mapping strategies. With this material mapping strategy, the percentage errors in computational reaction forces ranged from 40 to 749% in specimen 1, − 59 to 210% in specimen 2, 12 to 587% in specimen 3, − 44 to 292% in specimen 4, − 4 to 531% in specimen 5, 59% to 965% in specimen 6. For this material mapping strategy, specimens 1, 3, 5, and 6 had the lowest percentage errors of 40, 12, 4, and 59% respectively, when relationship 14 was used in the QCT-FEMs. Specimen 4 had a slightly lower percentage error of 36% using relationship 13 and specimen 2 had the lowest percentage error of 58% when relationships 1, 2, or 3 were used.

**Figure 4:**
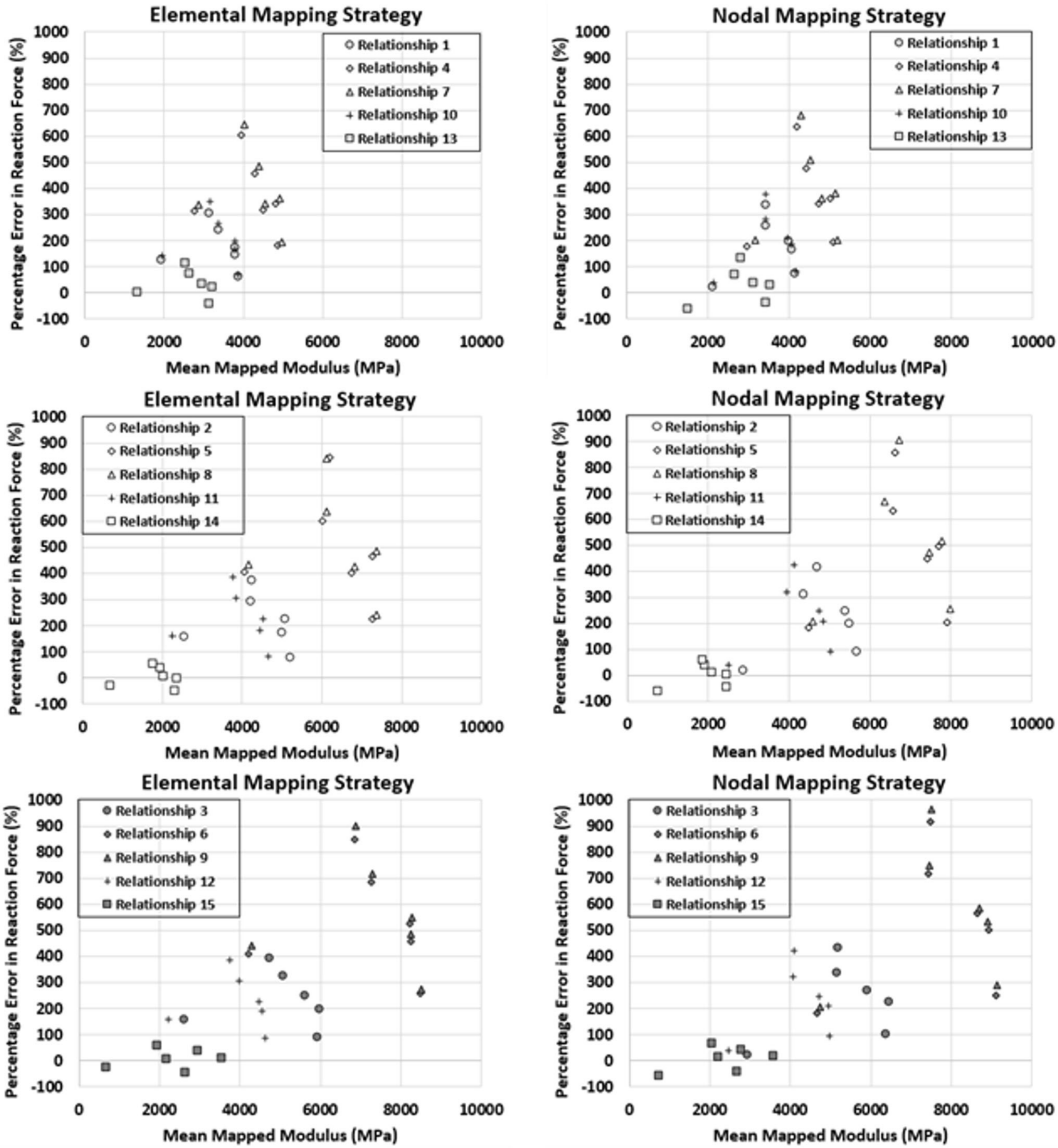
Percentage error plots between experimentally loaded scapular specimens and QCT-FEMs generated with fifteen different density–modulus relationships (Table 2). Relationships 1, 4, 7, 10, 13 use a transition between trabecular and cortical material mapping at 0.453 g_K2HPO4_/cm^3^ (1.0 g/cm^3^ apparent density), relationships 2, 5, 8, 11, 14 at 0.818 g_K2HPO4_/cm^3^ (1.8 g/cm^3^ apparent density). Relationship 15 uses a transition at 0.697 g_K2HPO4_/cm^3^ (1.54 g/cm^3^ apparent density), and relationships 3, 6, 9, and 12 use the trabecular density-modulus relationship extrapolated across the entire density range.

Comparing percentage errors in reaction force for each relationship and mean mapped modulus, the relationships that used a trabecular to cortical transition of apparent density of 1.0 g/cm^3^ (QCT equivalent density of 0.453 g_K2HPO4_/cm^3^) and associated trabecular and cortical material mapping showed overall lower mapped modulus than the remaining relationships (Fig. 4). The percentage errors using these density–modulus relationships were also lowest, with relationship 13 being best for both elemental and nodal material mapping. With a nodal material mapping strategy, comparable errors were observed with relationships 1 and 10. Relationships 4 and 7 had the highest mean mapped modulus and the highest percentage errors. When a trabecular to cortical transition at an apparent density 1.8 g/cm^3^ (QCT equivalent density of 0.818 g_K2HPO4_/cm^3^) and a uniform cortical modulus of 20,000 MPa was used, the mapped modulus increased for all relationships except relationship 14 (the Schaffler and Burr^25^ trabecular relationship). Similarly, this trabecular relationship had the lowest percentage errors and similar results were observed with lower percentage errors with relationships 2 and 11 (equivalent trabecular mapping to relationships 1 and 10) for nodal material mapping. Nearly identical results were observed when trabecular derived relationships were applied across the entire density range (relationships 3, 6, 9, 12) for both elemental and nodal material mapping. These relationships mapped the highest mean modulus and had the highest percentage errors in reaction forces.

## Discussion

This study compared density-modulus relationships and material mapping strategies used in QCT-derived finite element modeling (FEM) using DVC-driven boundary conditions (BCs). Using DVC-driven BCs allowed the QCT-FEMs to accurately replicate the experimental measured forces based on density–modulus relationship and material mapping strategy. There were large variations among the compared density-modulus relationships, with percentage errors in FEM reaction forces of up to 965%. Computational QCT-FEMs with the best material mapping were able to replicate the experimental forces to within 3% (relationships 13 and14) with elemental material mapping and within 4% (relationship 14) with nodal material mapping. There were only modest variations among specimens when either elemental or nodal material mapping strategies were used, indicating that either material mapping strategy can accurately replicate experimental loading of the scapula, provided an accurate density-modulus relationship is chosen.

This is important, because nodal material mapping can be easily implemented in custom-code used to generate QCT-FEMs and can easily be modified to account for partial volume effects (PVEs), as was done in the present study. Although with current FE-solvers these properties are generally assigned using field variables, nodal material mapping also allows for the mapping of heterogeneous distributions of materials in meshless models. At the micro-level, these models require significantly less computational resources and therefore allow for comparisons of very high-resolution models and/or non-linear models. This may be relevant at the continuum-level by allowing for larger model comparisons, especially those requiring larger computational resources such as those with contact or non-linear fracture and failure.

Generalized trabecular density-modulus relationships from pooled anatomic locations have been reported,^22^ and although not recommended, these relationships are often used in order to replicate material mapping in alternate anatomic locations because samples from multiple sites span a larger density range. This ignores the contribution of local trabecular morphology and its influence on trabecular modulus. In the present study, the trabecular density–modulus relationships used in 7, 8, 9, were developed from pooled anatomic sites and these relationships showed the greatest percentage errors in reaction forces for both elemental and nodal material mapping strategies. This may suggest that the local contribution of trabecular bone cannot be ignored in development of density-modulus relationships and that a generalized relationship for all anatomic sites is not possible. These relationships also mapped the highest modulus to the QCT-FEMs, providing QCT-FEMs that were much stiffer than the experimentally loaded specimens.

Similarly, the trabecular relationships 3, 6, 9, and 12, were developed using trabecular bone specimens, with the density range extrapolated to include cortical density mapping. As such, these relationships significantly overestimate the upper range modulus mapping and resulted in the highest percentage errors in reaction force (Fig. 4). Accounting for a transition of trabecular to cortical bone at an apparent density of 1.0 g/cm^3^ (relationships 1, 4, 7, 10, and 13) showed decreases in percentage errors for both elemental and nodal material mapping strategies. The relationships that used a mean cortical apparent density of 1.8 g/cm^3^ (relationships 2, 5, 8, 11, and 14) and a uniform modulus of 20,000 MPa for elements above this value, showed similar results to the extroplated trabecular density-modulus mapping relationships, except for relationship 14 which has the lowest percentage errors for most specimens depending on material mapping strategy. These results may suggest that trabecular density–modulus relationships accurately map the mechanical properties of the trabecular bone within the trabecular density range, but there needs to be more accurate cortical density–modulus relationships developed to accurately replicate the mechanical response of the cortical bone. Further investigation into these piecewise relationships are needed.

This may be confounded by conversions between density measures.^20^ Traditional density-modulus relationships are developed using bone cores mechanically loaded to derive an apparent modulus, which is related to each core’s mean apparent or ash density. Using these relationships to convert the QCT Hounsfield units into equivalent bone mineral density (BMD) and then into apparent or ash density for whole bones composed of both cortical and trabecular bone may introduce error in the FEM development process.^20^ The results of this study suggest that conversion to apparent density from QCT density can yield desired results (Table 3; Figs. 3 and 4); however, de-coupling the influence of density conversion, material mapping, density–modulus relationship, and trabecular/cortical piecewise transition could not be performed in the current study.

The trabecular relationships 1 to 6 were glenoid-specific.^18^ Interestingly, these relationships did not show the best agreement in replicating the experimental forces in these specimens. Although these relationships were developed using glenoid trabecular bone as an input, a relatively large tissue modulus was assumed in the models used to derive the density-modulus relationships (~ 10 GPa for relationships 1, 2, 3 and 20 GPa for relationships 4, 5 and 6). This fact may partially account for the overestimation in QCT-FEM loads when mapped with these relationships. Relationships 13, 14, 15 showed the lowest percentage errors in reaction force. The trabecular mapping used in these relationships provides the lowest modulus mapping of the trabecular bone (and least stiff models), indicating that at the whole-bone level, the true modulus is likely on the lower range of reported values. Although this trabecular relationship provided the closest reaction forces to experimental results, it overestimated the forces in specimens 1 and 6 and underestimated forces in specimens 2 and 4 when using both an elemental and nodal mapping strategy. This further indicates that the specimen-specific density distributions (Table 1 and Supplementary Materials) and the transitions between trabecular and cortical bone may play an important role in the accuracy associated with material mapping.

As assumed, applying varying constitutive relationships to map the mechanical properties of bone did not have a large effect on local displacement predictions generated by scapula QCT-FEMs. Regardless of the relationship selected, excellent agreement between the local experimental displacement measurements and QCT-FEM predictions were obtained, with both material mapping strategies. However, within the same models, large variations in reaction forces were observed. It has recently been suggested that local variations may be attributed to differences in bone micro-architecture^14^; however, the good agreement achieved with full-field displacements in the present study suggest that in QCT-FEMs this may not be true. Considering all density–modulus relationships had nearly identical full-field displacement linear regression results, further studies should be performed to elucidate the contributive variation in local mechanical properties of QCT-FEMs.

A strength of this study is that experimental boundary conditions were replicated in QCT-FEMs using DVC-driven boundary conditions. Replicating experimental boundary conditions has shown significant improvements in improving the accuracy of whole-bone QCT-FEMs,^14^,^15^ and have recently been reported as a main limitation in even the most robust studies that compare material mapping strategies and density-modulus relationships.^11^ The main limitation of this study is the small sample size. Due to the complexity associated with the experimental protocol required to generate DVC-derived BCs, the current study was limited to six specimens. However, the use of DVC-driven BCs along with local DVC measurements provided a highly-controlled experimental measure that allowed for the evaluation of multiple density-modulus relationships and material mapping strategies with high confidence that otherwise would not be possible.

This study compared density-modulus relationships and material mapping strategies of scapular QCT-FEMs with DVC-driven boundary conditions to experimentally loaded scapular models. It was found that elemental and nodal material mapping strategies are both able to accurately replicate experimental full-field displacements and reactions forces. Further investigation is required to determine the specimen-specificity of density-modulus mapping in scapular QCT-FEMs, the transition zone between trabecular and cortical material mapping and associated piecewise relationships, and whether improved cortical density-modulus relationship development improves linear-isotropic QCT-FEM accuracy.

## Electronic supplementary material

Below is the link to the electronic supplementary material.

Supplementary material 1 (PDF 189 kb)
