# Supplementary material for: Material Mapping of QCT-Derived Scapular Models: A Comparison with Micro-CT Loaded Specimens Using Digital Volume Correlation
Source: Ann Biomed Eng. 2019 Jul 11;47(11):2188–98. doi: 10.1007/s10439-019-02312-2 (PMC6838049; doi:10.1007/s10439-019-02312-2)
Supplement: Supplementary file 1 — Supplementary material 1 (PDF 189 kb) [file 10439_2019_2312_MOESM1_ESM.pdf]

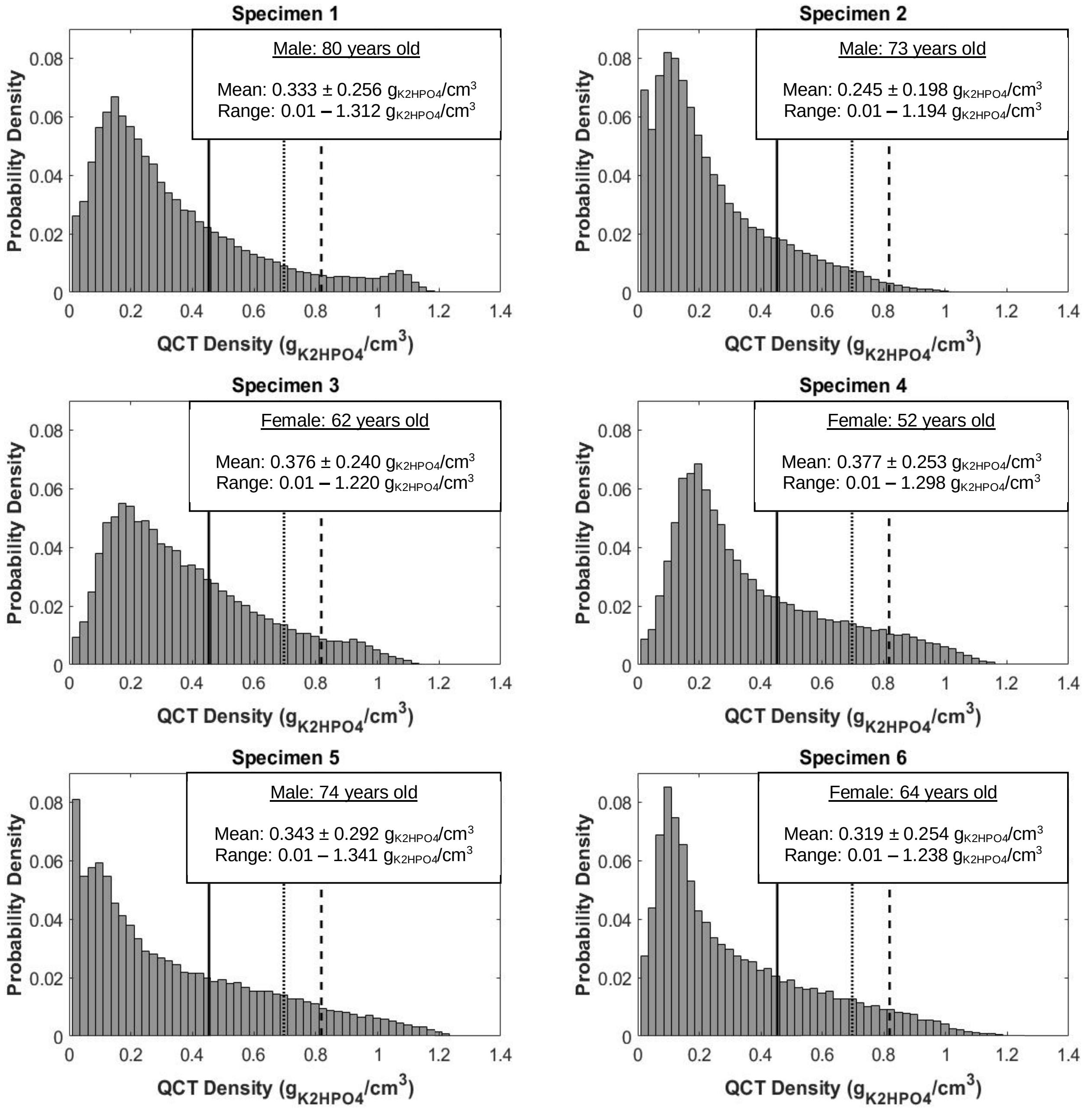

Figure: Histogram plots of the QCT density distribution in each of the six specimens. The lines represent transition between trabecular and cortical material mapping at 0.453 g_{K2HPO4}/cm^3 (relationships 1, 4, 7, 10, 14) (solid black line), 0.818 g_{K2HPO4}/cm^3 (relationships 2, 5, 8, 11, 14) (dashed black line), or 0.697 g_{K2HPO4}/cm^3 (relationship 15) (dotted black line).
